# A phase‐II randomized controlled pilot and feasibility study of nicotinamide riboside supplementation in older adults with amnestic mild cognitive impairment

**DOI:** 10.1002/alz70861_108851

**Published:** 2025-12-23

**Authors:** Christopher R Martens, Kevin P Decker, Theodore M DeConne, Faria Sanjana, Fiona Horvat, Nicholas A Rizzi, Catherine Awad, Elizabeth Habash, Joshua C Hobson, Michael L Armstrong, Nichole Reisdorph, Ryan T Pohlig, Alyssa M Lanzi, Curtis L Johnson, Matthew L Cohen, James M Ellison

**Affiliations:** ^1^ University of Delaware, Newark, DE USA; ^2^ Wake Forest University School of Medicine, Winston‐Salem, NC USA; ^3^ University of Colorado Anschutz Medical Campus, Denver, CO USA; ^4^ Thomas Jefferson University, Sidney Kimmel Medical College, Philadelphia, PA USA

## Abstract

**Background:**

The decline of nicotinamide adenine dinucleotide (NAD^+^) with aging may elevate risk of Alzheimer’s disease and related neurocognitive disorders (ADRD) by impairing cellular energy metabolism and reducing cerebral blood flow.

**Method:**

We conducted a 12‐week double‐blind, randomized, placebo‐controlled pilot study to evaluate the safety, tolerability, and preliminary efficacy of the NAD^+^ precursor, nicotinamide riboside (NR; 500 mg b.i.d.), for enhancing cognitive function and cerebral perfusion in older adults with mild cognitive impairment (MCI).

**Result:**

52 participants (33 females, 19 males) were randomized and 42 completed the trial (NR = 22, placebo = 20). Adherence was similar between groups (NR = 85 ± 20% vs. placebo = 91 ± 5%), with no serious adverse effects and comparable side effects. Blood NAD^+^ increased with NR (pre: 23.36 ± 1.94; post: 48.52 ± 4.12 µM) compared with placebo (pre: 24.12 ± 1.70; post: 25.14 ± 1.58 µM) with a significant treatment by time interaction (*p* <0.001). Cognitive function did not significantly improve, however, there was a modest increase in delayed recall memory from the WMS‐IV in the NR group (interaction *p* = 0.062). Perfusion of the left hippocampus increased with NR (pre: 51.7 ± 3.3; post: 58.0 ± 3.8 ml/min/100g) compared with placebo (pre: 55.6 ± 2.8; post: 51.7 ± 2.3 ml/min/100g; interaction *p* = 0.033); however, this was not associated with the change in memory performance. These outcomes were not strongly influenced by baseline plasma pTau‐217.

**Conclusion:**

NR is well‐tolerated in older adults with MCI and may enhance perfusion of the hippocampus regardless of underlying AD pathology. However, this was not associated with improved memory performance in this short‐duration trial. A larger phase‐III trial over a longer treatment duration is warranted to determine the efficacy of NR for delaying cognitive impairment due to ADRD.

